# Metabolic targeting of pantothenate by medium chain fats in glioblastoma cells

**DOI:** 10.1186/s12885-026-15909-3

**Published:** 2026-04-07

**Authors:** Erwann Pain, Pankaj K Singh, Yasin Khan, George Mears, Xiaoping Yang, Steven Lynham, Paul F. Devlin, Tricia Rutherford, Katie Lloyd Jones, Matthew C. Walker, Philip Beesley, Matthew S. Gentry, Robin SB Williams

**Affiliations:** 1 Centre for Biomedical Sciences, School of Biological Sciences, Royal Holloway University of London, Surrey, TW20 OEX Egham UK; 2 Department of Biochemistry & Molecular Biology, College of Medicine, University of Florida, University of Florida, Gainesville, FL USA; 3 Proteomics Facility, King’s College London, London, SE5 9NU UK; 4 Department of Biological Sciences, Sustainable Engineering and Food Security, Royal Holloway University of London, Surrey, TW20 OEX Egham UK; 5 Clinical Nutrition, Vitaflo International Ltd, Liverpool, UK; 6 Department of Clinical and Experimental Epilepsy, Institute of Neurology, University College London, London, WC1N 3BG UK; 7 Centre for Biomedical Sciences, Royal Holloway University of London, Surrey, TW20 OEX UK

**Keywords:** Cancer, Decanoic acid, Fatty acid metabolism, Glioblastoma, Glycolysis, Medium chain triglycerides (MCT), Metabolic, Pantothenate

## Abstract

Ketogenic diets are commonly used for epilepsy therapy and are increasingly being considered in cancer treatment, where metabolic reprogramming triggered by glucose restriction and ketosis alters energy provision as the therapeutic mechanism. However, a recently developed medium chain triglyceride product, with a high decanoic acid (DA) to octanoic acid (OA) ratio (DA: OA) has enabled a much less restrictive diet and is clinically effective as an epilepsy treatment, yet no studies have explored the product in cancer models. Here we investigate metabolic mechanisms of DA, OA and DA: OA using a cellular glioblastoma model, focusing on changes in energy metabolism. We show, at a transcriptional level, that DA provides the dominant regulatory influence, enhancing transcription of fatty acid and amino acid metabolism pathways, while reducing cancer-associated glucose metabolism and kinase pathways. DA: OA treatment shared some of these effects, with little influence from OA. At a protein level, DA: OA treatment played the dominant role, regulating metabolic, cancer and signalling pathways, including a large network centred around albumin and protein kinase activities. Finally, at a metabolomic level, both DA and DA: OA treatment reduced vitamin B5 (pantothenate) levels, which is necessary for the synthesis of coenzyme A used in the activation of fatty acids for energy provision, suggesting a novel mechanism to reduce cellular energy provision in cancer. Importantly, these effects occur under high glucose conditions. Thus, we show that decanoic acid-rich treatments may regulate glioblastoma cell biology through targeted effects on glucose and fatty acid metabolism and kinase signalling and may trigger a potential reduction in pantothenate levels in glioblastoma cells, providing a potential novel therapeutic mechanism in cancer treatment.

**Supplementary Information:**

The online version contains supplementary material available at 10.1186/s12885-026-15909-3.

## Introduction

Ketogenic diets are widely used in the treatment of epilepsy and are increasingly being investigated for other diseases including cancer, but further studies are needed to clarify molecular mechanisms [1, 2]. These diets involve increasing fat intake and reducing carbohydrate consumption. The initial form of the diet, called the ‘classical’ ketogenic diet, requires a very low carbohydrate intake with around 90% of energy derived from fats [3]. This diet triggers a reduction in blood glucose leading to altered energy metabolism and the production of ketones through fatty acid oxidation to provide energy to the brain [4]. Alternatively, a less stringent medium chain triglyceride (MCT) diet comprising medium chain fatty acids (MCFA), decanoic and octanoic acid in a 40:60 ratio, enables greater consumption of carbohydrates whilst maintaining ketosis via octanoic acid [5], and is clinically effective in drug-resistant epilepsy treatment [6]. More recently, a new MCT diet has been introduced comprising an enhanced ratio of decanoic and octanoic acid (DA: OA; 80:20), that also provides seizure control [K.vita; 7], but surprisingly does not require glucose restriction or the resulting ketosis, raising questions about the role of ketones as the therapeutic mechanism of the MCT diet. However, the effects of this diet on metabolism and energy provision in cancer models have not been investigated.

Ketogenic diets are increasingly being used in cancer treatment, where the diets are generally well tolerated, feasible and safe [1]. These diets show antitumor effects in 60% of studies, mostly on glioblastoma models, with a reduction in glucose levels, induction of ketosis, and improvements in quality of life. However, reduced glucose uptake or a reduction in tumour size may not correlate with glucose or ketone levels [7, 8]. Medium chain fatty acids have been suggested to have potential efficacy in cancer treatment [9], or as an additional supplement to classical ketogenic diets in cancer studies [10].

Beyond carbohydrate restriction and ketosis for energy provision, several molecular mechanisms have been identified for both the classical and MCT ketogenic diets that may impact cancer development [11, 12]. Both diets enhance mitochondrial load to stabilise energy provision [13–15]. Specific mechanisms for MCFAs have been demonstrated without glucose restriction, where decanoic acid specifically inhibits AMPA receptors [16] and mTORC1 activity [17]. Classical ketogenic diets have also been shown to function through metabolic effects in animal models, regulating energy metabolism at a transcriptional level [13]. However, few studies have investigated metabolic roles for MCFAs, alone or in combination, in the regulation of cancer cell function.

In this paper we investigate the role of individual MCFAs and the clinically effective blend of these as DA: OA [18] on cancer cell energy metabolism, in the absence of carbohydrate restriction. We show that decanoic acid and DA: OA treatments provide a dominant transcriptional role in the downregulation of glucose metabolism and cancer pathways and the upregulation of fatty acid metabolism pathways. Similar glucose metabolism and cancer pathway downregulation was observed at a protein level, in addition to the identification of a large signalling network involving albumin, kinase pathways and glutathione-related proteins. Surprisingly, we also identify a key metabolic effect of decanoic acid and DA: OA in the reduction of pantothenate (B5) levels necessary for fatty acid metabolism, providing a potential novel therapeutic mechanism in cancer treatment.

## Methods

### Chemicals

Decanoic and octanoic acid were purchased from Alfa Aesar (A14788, A11149 respectively). The fatty acids were dissolved in dimethylsulphoxide (DMSO) at 150 mM (VWR, MFCD00002089).

### Cell culture

TheU251 MG human cell line was obtained from Sigma Ltd (cat. 9063001). Cells were stored in liquid nitrogen and taken from storage to be grown for no more than 10 passages. Cells were cultivated at 37 °C, 5% CO_2_ in either 25 cm^2^ or 75 cm^2^ cell culture flasks (Corning, 430639/430641U) in Minimum Essential Medium Eagle (Merck, M4655, 5.6 mM glucose) with non-essential amino acids (Merck, M7145), Sodium pyruvate (Merck, S8636), 10% Foetal Bovine Serum (Merck, F7524) and 1% Penicillin/Streptomycin (Life Technologies Corp, 15140-122), following manufacturer’s instructions. At 80% confluency, cells were trypsinised (ThermoFisher, 25300054) and split to be seeded at 3.10 × 10^4^cells/cm^2^ in a new flask.

### RNA sequencing

U251 cells in T25 flasks were treated with either 150 µM decanoic or octanoic acid, 120/30 µM decanoic/octanoic acid dissolved in DMSO for 4 days. The control group was treated with DMSO only (0.1% final concentration). Media was changed every 48 h. The 120/30µM group was designed to be treated with an 80:20 ratio of decanoic: octanoic acid, reproducing the proportion of MCT used in supplements in feasibility study on drug resistant epilepsy [18]. Total RNA from three biological replicates was extracted using the RNeasy kit (Qiagen, 74104), with mRNA purification from total RNA using magnetic bead, and the obtained samples were fragmented in 50 bp mRNA fragments. cDNA was synthesized from mRNA fragments ligated with adapters, amplified using real-time PCR and sequenced on an Illumina platform by Novogene. Raw reads were cleaned by removing reads containing adapter, read containing poly-N and low-quality reads using fastp software. Reads were aligned to a known genome using Hisat2, and quantification of genes expression was carried out using FeatureCounts. Differential gene expression was assessed by using the DESeq2 R package, and differential pathway analysis was carried out using Gene Set Enrichment Analysis (GSEA, http://www.broadinstitute.org/gsea/index.jsp) with the Kyoto Encyclopedia of Genes and Genomes (KEGG) database (https://www.genome.jp/kegg/).

### Quantification of metabolic profile by gas chromatography mass spectrometry

U251 cells were grown and treated as for RNA sequencing. Cells were washed twice with dPBS (ThermoFisher, 14190144), and extracted with 1 ml on 50% ice cold methanol using a cell scraper (ThermoFisher, 179693PK), transferred to 1.5 ml Eppendorf tubes, and combined with 1 µL 20 mM L-norvaline (Merck, N7627), vortexed, incubated on ice for 30 min. Following incubation, the samples were centrifuged at 15,000 rpm for 10 min at 4 °C to separate polar metabolites (supernatant) from protein/lipids (pellet). The polar phase was immediately frozen in liquid nitrogen and stored in -80 °C. The protein fraction was hydrolysed in 100 µL 6 N HCl (heat at 95 °C for 2 h). The reaction was quenched by adding 200 µL of 100% methanol containing 200 µM L-norvaline. The samples were vortexed thoroughly and centrifuged again at 15,000 rpm for 10 min at 4 °C. The supernatant, containing digested proteins, glycogen, and glycans, was transferred to a separate tube. Both polar and protein fractions were dried for 2–4 h. For derivatization, 80 µL of MSTFA (Thermo Scientific, TS-48915) was added to each sample, which were then incubated at 37 °C for 30 min with thorough mixing.

The samples were analysed using gas chromatography-mass spectrometry (GC-MS) in full scan mode, performed on an Agilent 7800B gas chromatograph coupled to a 5977B single quadrupole mass spectrometer, using 1 µL aliquots injected for analysis. The method adapted protocols [19, 20], with a modified GC temperature gradient: an initial temperature of 130 °C, held for 4 min, followed by a rise of 6 °C/min to 243 °C, then 60 °C/min to 280 °C, held for 2 min.

Batch data were processed using the Data Extraction for Stable Isotope-labelled Metabolites (DExSI) software package. Metabolite identification was performed by deconvoluting the mass spectra using the Automated Mass Spectral Deconvolution and Identification System (AMDIS) software [21]. The relative abundance of metabolites was first normalized to L-norvaline to account for sample loss, and then to protein input based on amino acid content in the pellet fraction. Analysis of metabolic pathways employed definition by the KEGG resources.

### Quantification of proteomic profile by liquid chromatography mass spectrometry

U251 cells were grown and treated as for RNA sequencing. Cell lysates were prepared using RIPA buffer and protease cocktail inhibitor (ThermoFisher, 89900) and precipitated with cold acetone protocol. Protein pellets were resuspended in 100 mM triethylammonium bicarbonate buffer (TEAB) and tryptic digested at a ratio of 100:1 (protein : trypsin) at 37 °C for overnight, after being treated with Dithiothreitol (DTT) reagent for reduction followed by iodoacetamide (IAA) alkylation. The digested peptides were desalted with C18 spin column (Pierce C18 spin column, ThemoFisher 89873, each containing 8 mg) and dried by SpeedVac prior (Thermo SAVANT SPD131DDA) following manufacturer’s instructions, to enable LCMS analysis.

The tryptic peptides were directly ionized within the Easy-spray ion source (ThermoFisher) and injected into Orbitrap Eclipse Tribrid mass spectrometry (ThermoFisher) coupled with Ultimate 3000 RSLC nano system for analysis. For liquid chromatography, a reverse phase Thermo Acclaim Pepmap trap column (2 cm length, 75 μm in diameter and 3 μm C18 beads) were connected to the nanoflow HPLC on an Easy-spray C18 nano column (50 cm length, 75 μm in diameter, ThermoFisher). Buffer A (0.1% formic acid in water) and buffer B (80% ACN, 0.1% formic acid) were used. Peptides were eluted with a 60 min gradient, ramping from 4% to 10% to 5 min, from 10% to 30% to 37.5 min, from 30% to 40% to 40 min, from 40% to 99% to 42.5 min, keep at 99% to 46.9 min and decreasing to 4% at 47 min.

The MS instrument was operated in the positive ion mode with an electrospray through a heated ion transfer tube at 275 °C. MS DIA datasets were acquired within Xcalibur 4.7 using the following parameters: scan range 400-900 m/z, MS resolution of 60,000 at m/z 200, a normalized AGC target 250%, and maximum injection time of Auto. The MS/MS scan was performed in HCD mode with the following parameters: using 12 Da isolation window with 1 Da overlap over 400–900 *m*/*z* precursor and scan range of 145–1450 *m*/*z*, orbitrap resolution 15,000 with maximum injection time of Auto, a normalized AGC target 800%, and normalized collision energy 30%. All data were acquired in centroid mode. The mass spectrometry proteomics data have been deposited to the ProteomeXchange Consortium via PRIDE [22], dataset identifier PXD068251.

Resulting DIA raw files were searched against the Uniprot/Swiss-Port database (Human) following analysis pipeline within PEAKS Studio software (Bioinformatics Solutions Inc, version 12). DIA DB search parameters: precursor and fragment mass error tolerances (auto detected) with match between run, trypsin as enzyme with 1 miss cleavage, cysteine carbamidomethylation as a fixed modification, peptide filter FDR 1%. Label-free quantification was applied here with DIA LFQ workflow embedded, using high precision mode, default filter setting for peptide and protein and TIC normalization. The extracted precursor ion intensities were applied for downstream differential analysis.

### Statistical analysis

All statistical analyses were performed using GraphPad Prism (version 10.3; GraphPad Software, San Diego, CA, USA), with all numerical data presented as mean ± SD. Differential expression analysis was performed using a Wald test with the DESeq2 R package (1.20.0). Adjusted P values (Benjamini and Hochberg’s) of ≤ 0.05 were assigned as differentially expressed. Gene Ontology (GO) enrichment analysis of differentially expressed genes employed the clusterProfiler R package for statistical enrichment of differential expression in KEGG pathways and presented as mean ± SEM. For proteomic analysis (four independent samples), comparisons between control and each treatment used two-way ANOVA analysis with Dunnett’s multiple comparison adjustment (*P* < 0.05). For metabolomic analysis, two-way ANOVA multiple comparisons with Dunnett’s multiple comparison test were used on the entire data set, with at least three independent samples providing data on the target protein for all treatments.

## Results

To investigate the molecular mechanisms of MCFAs in regulating cancer cell energy metabolism, we employed a human astrocyte-derived glioblastoma (GBM) cell line (U251), previously used as a cancer model for fatty acid metabolism studies [23, 24]. We employed fatty acid concentrations that reflect those found in epileptic patients during treatment [18] (150 µM final concentration), over 4 days to enable metabolic changes as shown in human studies [25]. We initially investigated the effects of fatty acids on energy metabolism relevant to GBM growth using transcriptomics [26, 27].

**Fig. 1:**
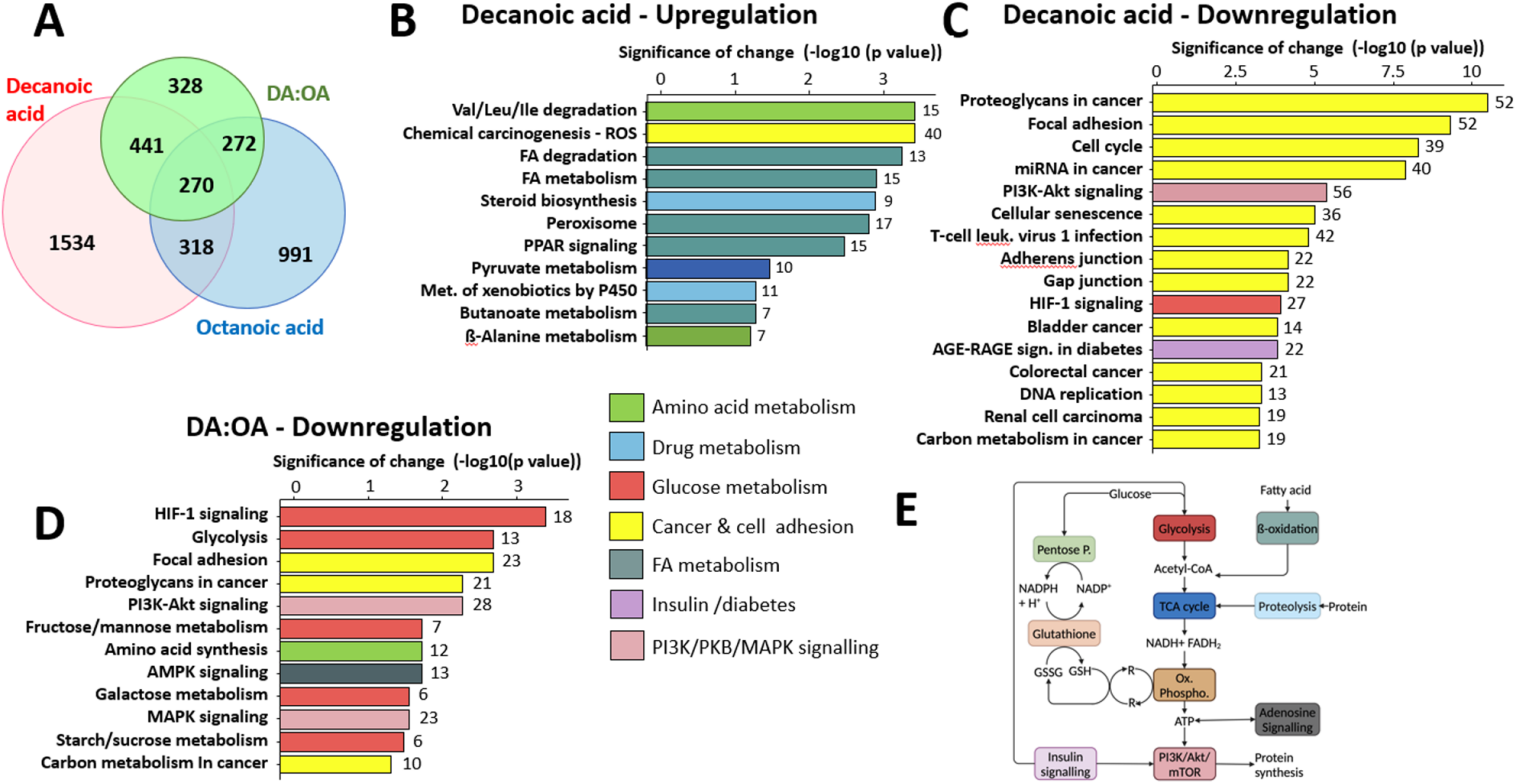
Transcriptomic analysis of GBM cells following MCFA treatment. GBM cells were treated with decanoic acid or octanoic acid (150 μM, *N* = 3) or a Decanoic Acid Rich Supplement (DA:OA: 120 μM decanoic acid and 30 μM octanoic acid, *N *= 3) for 4 days, and transcriptomic analysis identified significantly altered gene expression. **A** Venn diagram analysis of genes showing significantly altered expression levels following treatment. **B-D** Analysis of significantly altered genes in distinct KEGG pathway (-log10(p value)) are shown with significance threshold of *p* < 0.05, and KEGG pathways grouped by colour coding. Numbers above each bar are the number of genes modified in the pathway. **E** Schematic of specific pathways involved in energy metabolism linking glucose and fatty acid metabolism, and related pathways. P values are provided in Supplementary Fig. S2-3

Following treatment of GBM cells with all three MCFAs (decanoic, octanoic and DA: OA treatment), no significant change was observed in cell viability after 4 days. However, incubation with the MCFAs induced a broad change in transcription. Analysis identified 4,172 genes significantly modified in any condition (Fig. 1A), with 6.5% (270 genes) altered in all three conditions suggesting that many changes were fatty acid specific. Decanoic acid only treatment regulated the largest proportion of genes (63%, 2626 genes) with 60% unique to decanoic acid. Octanoic acid treatment modified the expression of 45% (1869 genes), where 52% of these genes were unique to octanoic acid. Decanoic and octanoic acid treatment shared only 17%, suggesting that the mechanisms of action of these fatty acids on transcription were distinct. Interestingly, DA: OA treatment significantly modified the expression of 31% of these genes, of which 75% (983 genes) shared an effect with either decanoic or octanoic treatment conditions, with 328 unique genes altered (25%). Thus, decanoic and octanoic acid treatment provided mainly distinct effects on gene transcription, and DA: OA treatment shared most of the effect with one or the other fatty acid but also provided specific alterations of gene transcription.

Analysis of transcriptional regulation following MCFA treatment then focused on significantly altered metabolic and disease pathways identified using the KEGG database. Decanoic acid treatment significantly upregulated 15 KEGG pathways (Fig. 1B) and downregulated 75 (Fig. 1C, Supplementary Fig. S1). Of the 15 most significantly upregulated pathways, five were involved in fatty acid metabolism and signalling (including fatty acid degradation *p* < 0.001; fatty acid metabolism *p* < 0.001; peroxisome *p* = 0.001; PPARγ *p* = 0.002; butanoate metabolism *p* = 0.033), two were involved in amino acid metabolism (Valine, leucine, isoleucine degradation *p* < 0.001; ß-alanine metabolism *p* = 0.04), and two in drug metabolism (steroid biosynthesis *p* < 0.001; Metabolism of xenobiotics by cytochrome P450 *p* = 0.03). Of the 20 most significantly down-regulated pathways by decanoic acid (Fig. 1C), 13 were related to cancer and cell adhesion, one to glucose metabolism (HIF-1 signalling *p* < 0.001) and one relating to protein kinase function (PI3K-AKT *p* < 0.001).

Following DA: OA treatment, 14 pathways were downregulated, including several related to cancer and cell adhesion pathways common to decanoic acid treatment (focal adhesion *p* = 0.001; proteoglycans in cancer *p* = 0.003; central carbon metabolism in cancer *p* = 0.02) (Fig. 1D), as well as five pathways involved in glucose and related compound metabolism (HIF-1 signalling *p* < 0.001; glycolysis *p* = 0.001; fructose/mannose metabolism *p* = 0.011; galactose metabolism *p* = 0.016; starch/sucrose metabolism *p* = 0.02), and three pathways in kinase signalling (PI3K-AKT signalling *p* = 0.003; MAPK signalling *p* = 0.02 and AMPK signalling *p* = 0.01). Only one pathway was significantly upregulated by DA: OA treatment (Supplementary Fig. S1). Octanoic acid treatment provided relatively few significant changes, with 10 KEGG pathways significantly upregulated and four downregulated (Supplementary Fig. S1). This analysis suggests a dominant role for decanoic acid-containing treatments in energy metabolism in GBM cells relating to cancer, sugar metabolism and kinase pathways, thus further analysis of energy metabolism pathways was undertaken (Fig. 1E).

### MCFA transcriptional regulation of glucose uptake and glycolysis

**Fig. 2:**
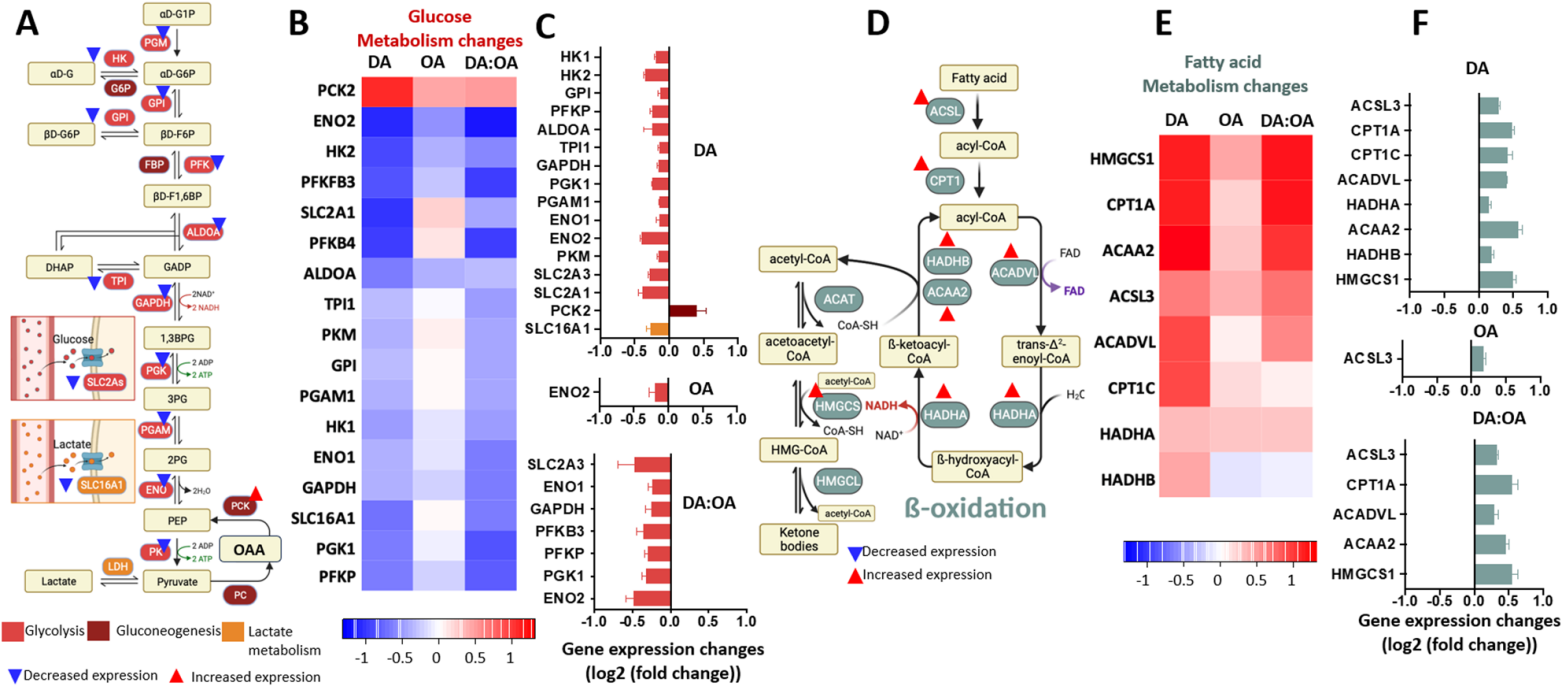
Analysis of transcription changes in Oxidative Phosphorylation related genes in GBM cells following MCFA treatment: **A** Schematic of oxidative phosphorylation, showing electron transfer from NADH and FADH_22_ through the respiratory complexes and the associated proton pumping at complexes I, III, and IV. The resulting proton motive force across the inner mitochondrial membrane drives ATP synthesis. **B** Heatmaps of significant changes in gene expression in oxidative phosphorylation genes following treatment with decanoic acid(150 μM), octanoic acid (150 μM) or DA:OA (120:30 μM decanoic: octanoic acid), from 3 independent samples (*n* = 3), showing genes with significant alteration of expression in at least one of the treatment conditions where expression changes are represented as log2(fold change), where a value of 1 corresponds to a doubling of expression and − 1 corresponds to a halving. **C** Genes involved in oxidative phosphorylation that were significantly up or downregulated were compared between each treatment condition, with gene expression change in represented in log2(fold change). **D** Schematic of the pentose phosphate pathway (PPP), glucose is metabolised to produce energy by formation of NADPH. **E** Genes involved in PPP and **F** relating to mTOR, insulin and adenosine signalling. Figure C, E and F represent the difference in gene expression between the control and treated conditions for each individual gene

Specific transcriptional changes were analysed relating to sugar transport, glycolysis and gluconeogenesis (Fig. 2A). Heat map comparison of significantly altered gene expression (Fig. 2B) indicated common strong effects following decanoic acid and DA: OA treatments, with reduced effects following octanoic acid treatment. Comparison of specific gene expression changes (Fig. 2C) indicated that decanoic acid treatment significantly decreased expression for ten of eleven genes encoding enzymes involved in glycolysis (Fig. 2A, C). These included both astrocytic and neuronal glucose transporter 1 and 3 (SLC2A1 and SLC2A3 which encode GLUT enzymes to maintain glucose levels in the brain (*p* = 0.005) [28, 29]; hexokinase enzymes (HK) (*p* = 0.009) which catalyse the first rate-limiting first step of glycolysis [30] and SLC16A1, coding for MCT1, a transporter involved in transport of both lactate and ketones [31]. Interestingly expression of phosphoenolpyruvate carboxykinase (PCK2) increased, a gene that controls the rate limiting step in gluconeogenesis and tricarboxylic acid (TCA) cycle flux [32]. Thus, only decanoic acid and DA: OA treatment regulated the expression of genes controlling glucose metabolism, reducing this pathway.

### MCFA transcriptional regulation of fatty acid metabolism

Specific transcriptional changes were analysed relating to fatty acid metabolism (Fig. 2D) to indicate a decanoic- and DA: OA-dependent upregulation of genes controlling ß-oxidation and acyl-CoA formation (Fig. 2E, F). Decanoic acid treatment significantly increased expression of key genes involved in fatty acid degradation (ACADVL, ACAA2, HADHA, HADHB) (Fig. 2F), two genes in acyl-CoA formation (ACSL3, ALDH3A2), and two genes relating to fatty acid transport into the mitochondrion (CPT1A and CPT1C). Many of these effects were also shown following DA: OA treatment including two genes involved in acyl-CoA formation and transport into the mitochondrion (ACSL3, CPT1A) and three genes involved in ß-oxidation (ACAA2, ACADVL) as well as HMGCS1 (Fig. 2F). In contrast, octanoic acid significantly upregulated one gene, ACSL3, involved in the synthesis of fatty acyl-CoA, a substrate of the ß-oxidation (Fig. 2F). Thus, decanoic acid-containing treatments provided a significant increase in expression of fatty acid metabolic enzymes, an effect not observed following octanoic acid treatment.

### MCFA transcriptional regulation of TCA cycle, oxidative phosphorylation

**Fig. 3:**
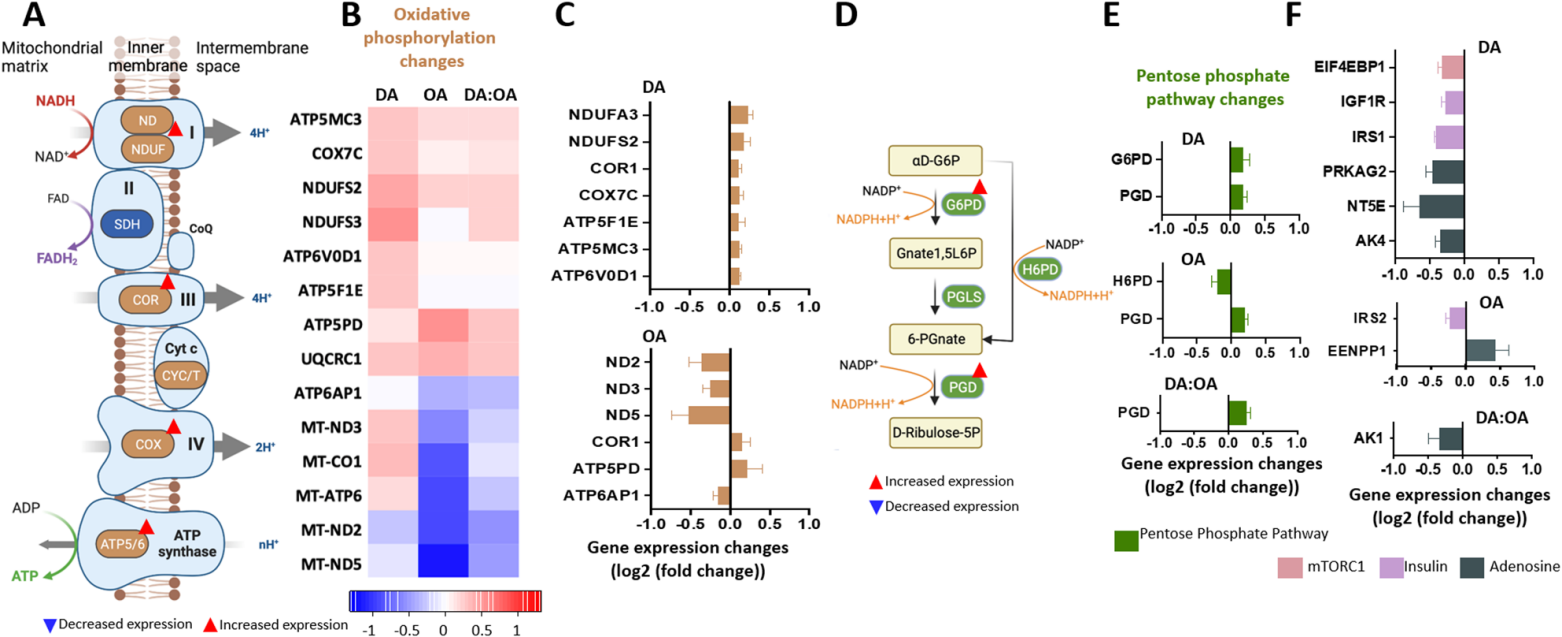
Analysis of transcription changes in Oxidative Phosphorylation related genes in GBM cells following MCFA treatment: **A** Schematic of oxidative phosphorylation, where complex I, II, and III pump protons from NADH and FADH_2_ in the mitochondrial intermembrane space, creating an electrochemical proton gradient. ATP synthase uses the energy provided by this gradient to synthesise ATP. **B** Heatmaps of significant changes in gene expression in oxidative phosphorylation genes following treatment with decanoic acid (150 μM), octanoic acid (150 μM) or DA:OA (120:30 μM decanoic: octanoic acid), from 3 independent samples (*n* = 3), showing genes with significant alteration of expression in at least one of the treatment conditions where expression changes are represented as log2(fold change), where a value of 1 corresponds to a doubling of expression and − 1 corresponds to a halving. **C** Genes involved in oxidative phosphorylation that were significantly up or downregulated were compared between each treatment condition, with gene expression change in represented in log2(fold change). **D** Schematic of the pentose phosphate pathway (PPP), glucose is metabolised to produce energy by formation of NADPH. **E** Genes involved in PPP and **F** relating to mTOR, insulin and adenosine signalling. Figure C, E and F represent the difference in gene expression between the control and treated conditions for each individual gene

Specific transcriptional changes were analysed relating to energy metabolism downstream of glycolysis and fatty acid metabolism including pyruvate dehydrogenase (PDH), the TCA cycle (Supplementary Fig. S4) and oxidative phosphorylation (Fig. 3A). An increased expression of the gene encoding for the PDHB subunit of PDH was identified, which is involved in the synthesis of acetyl-CoA together with two genes encoding components of the TCA cycle, IDH1 involved in the oxidation of isocitric acid to α-ketoglutarate [33], and ACLY, responsible for the reverse of the first step of the TCA cycle, producing acetyl-CoA from citric acid. In oxidative phosphorylation (Fig. 3A), heatmap analysis indicated relatively small changes compared to glycolysis and fatty acids metabolism, with different effects of the three treatments (Fig. 3B, C). Decanoic acid treatment provided a consistently small increase in expression of oxidative phosphorylation-related genes for seven genes including mitochondrial respiratory chain complex I, complex III, and complex IV, and three genes relating to ATP synthase activity (ATP6V0D1/5MC3/5F1E) (Fig. 3B, C). Octanoic acid treatment caused an even smaller increase or decrease in expression of these genes. Interestingly, DA: OA treatment did not alter the expression of any genes in oxidative phosphorylation, likely through opposing effects of both fatty acids. In summary, decanoic acid treatment provided a small upregulation of genes involved in oxidative phosphorylation, and effect not observed with either octanoic acid or DA: OA treatment (Fig. 3B, C).

## MCFA transcriptional regulation of pentose phosphate pathway, adenosine and insulin signalling, mTORC1 and NMDA changes

Transcriptional changes in several genes related to the potential therapeutic mechanisms of dietary treatment were also examined including the pentose phosphate pathway (PPP), adenosine, insulin, and mTORC1 signalling [11, 15, 17] (Fig. 3D, E). Decanoic acid increased expression of two PPP-related genes (G6PD and PGD) involved in NADPH synthesis [34], with the latter also increased by DA: OA treatment (Fig. 3.B). In contrast, octanoic acid increased PGD and decreased H6PD gene expression. Interestingly, decanoic acid treatment also reduced expression of mTORC1 (EIF4EBP1) and insulin related genes (IRS1 and IGF1R) potentially relating to anti-cancer mechanisms [35]. Decanoic acid treatment also reduced the expression of several adenosine-regulation related genes implicated in cancer progression [36], including NT5E, which is involved in the dephosphorylation of AMP to adenosine, the nutrient sensor AMPK regulatory subunit (PRKAG2), and two adenosine kinase isoforms that catalyse production of ADP from ATP and AMP.

## Decanoic Acid and DA: OA proteomic regulation

**Fig. 4:**
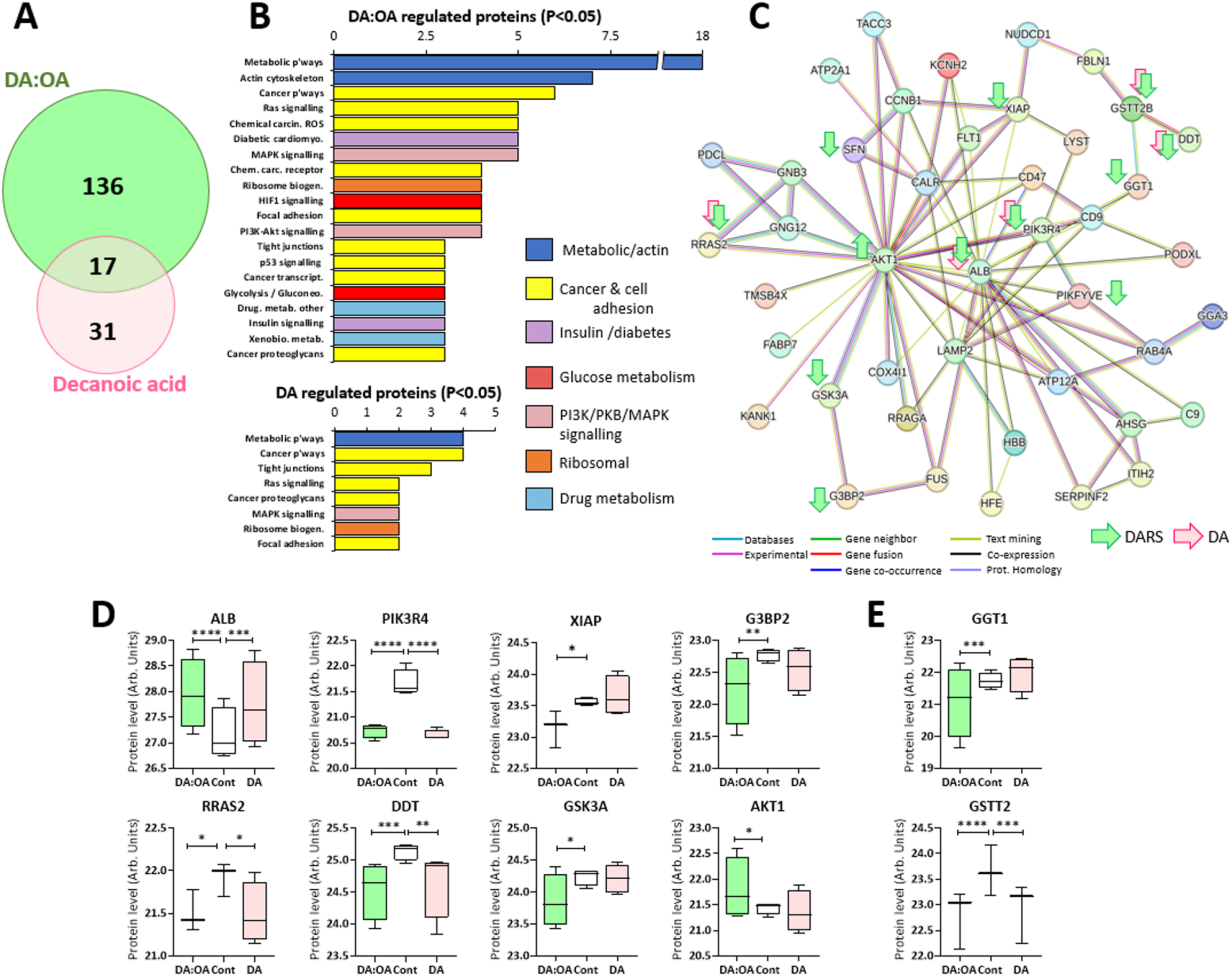
Global proteome changes GBM cells following MCFA treatment identified regulated pathways and networks. Cells were treated with decanoic acid (150 µM) or DA:OA (120:30 µM decanoic: octanoic acid) at a total concentration of 150 µM, for 4 days, and protein extracts were analysed by LC-MS and quantified using PEAKS from four independent samples (*n* = 4). Statistical analysis of the whole protein data set employed 2-way ANOVA with Dunnett’s correction, with each protein identified at least 3 times. **A** DA: OA treatment provided the largest number of significant changes in protein levels. **B** KEGG pathway analysis of significantly changed proteins provided specific pathways regulated by both treatments and associated with different aspects of cancer biology. STRING analysis identified network of significantly changed proteins following DA:OA (green arrow) and Decanoic acid (red arrow) treatment (Suppl. Fig x): **C** a large network centred around Albumin (ALB) and PI3K/AKT signalling, and including glutathione function, where highly significant changes (*P* < 0.001) are indicated with increased or decreased levels for each protein and treatment; **D** Quantification of protein level changes in key kinase and cancer-related proteins, and **E** proteins in glutathione regulation.* *P* < 0.05, ** *P* < 0.01, ***P < 0.001, ****P < 0.0001

Since the effect of MCFAs on transcription was primarily through decanoic acid and DA: OA treatment in GBM cells, further analysis investigated these treatments on global cytosolic protein levels. In these experiments, treatment conditions reproduced those used in the transcriptomic studies, and LC-MS analysis identified 3,345 individual proteins. From this analysis, 184 showed significant changes with one or both treatments (Fig. 4A, Supplementary Fig S5 and S6), with DA: OA treatment surprisingly providing the greatest number of significant changes (74%). KEGG analysis identified that these changes were related to metabolic and cancer-specific pathways (Fig. 4B), including diabetes and insulin, MAPK and PI3K, glucose metabolism and ribosomal biogenesis. Thus, proteomic analysis confirms similar pathway regulation for MCFAs as shown in transcriptomic analysis.

STRING analysis of significantly changed proteins identified a single large network centred around elevated albumin levels, several kinase enzymes, and glutathione (Fig. 4C) (Supplementary Fig S7). Quantification of individual protein changes within the network (Fig. 4D) showed both treatments increased albumin levels associated with fatty acid uptake [37], and reduced levels of PIK3R4, a regulatory subunit of phosphatidylinositol 3-phosphate that is upregulated in several cancers [38]. Other cancer-associated proteins reduced by both treatments were RRAS, a key oncogene GTPase overactivated in various cancers [39], and DDT, D-dopachrome decarboxylase, that is highly expressed in a range of cancers and drives growth [40]. DA: OA treatment alone decreased levels of XIAP, an E3 ubiquitin-protein ligase [41], and G3BP2, a Ras-GAP SH3 domain binding protein that is upregulated in many cancers to promote [42] and promotes tumour growth and survival.

Other regulated kinases include a reduction in GSK3A levels, where the protein plays a key role in insulin signalling [43] and promotes tumorigenesis [44], and AKT1, that is widely demonstrated to be a target for cancer treatment, but activation may benefit late-stage cancer treatment [45]. Finally, two glutathione level regulators GGT1 (Glutathione hydrolase 1 proenzyme) and GSTT2 (Glutathione S-transferase theta-2) were reduced, where glutathione plays an important role in many cancers and elevated levels are associated with enhanced proliferation [46], and reduced levels are likely to enhance cancer cell oxidative stress. Tissue expression analysis of proteomic changes also suggests significant potential in regulating leukaemia cell function (Supplementary Fig. S8). Thus, proteomic analysis identified a range of similar responses to those shown in transcriptional mechanisms and highlighted a protein network regulated by DA: OA and decanoic acid treatment including a range of effects that may be linked to cancer treatment.

## Decanoic acid and DA: OA metabolomic regulation

**Fig. 5:**
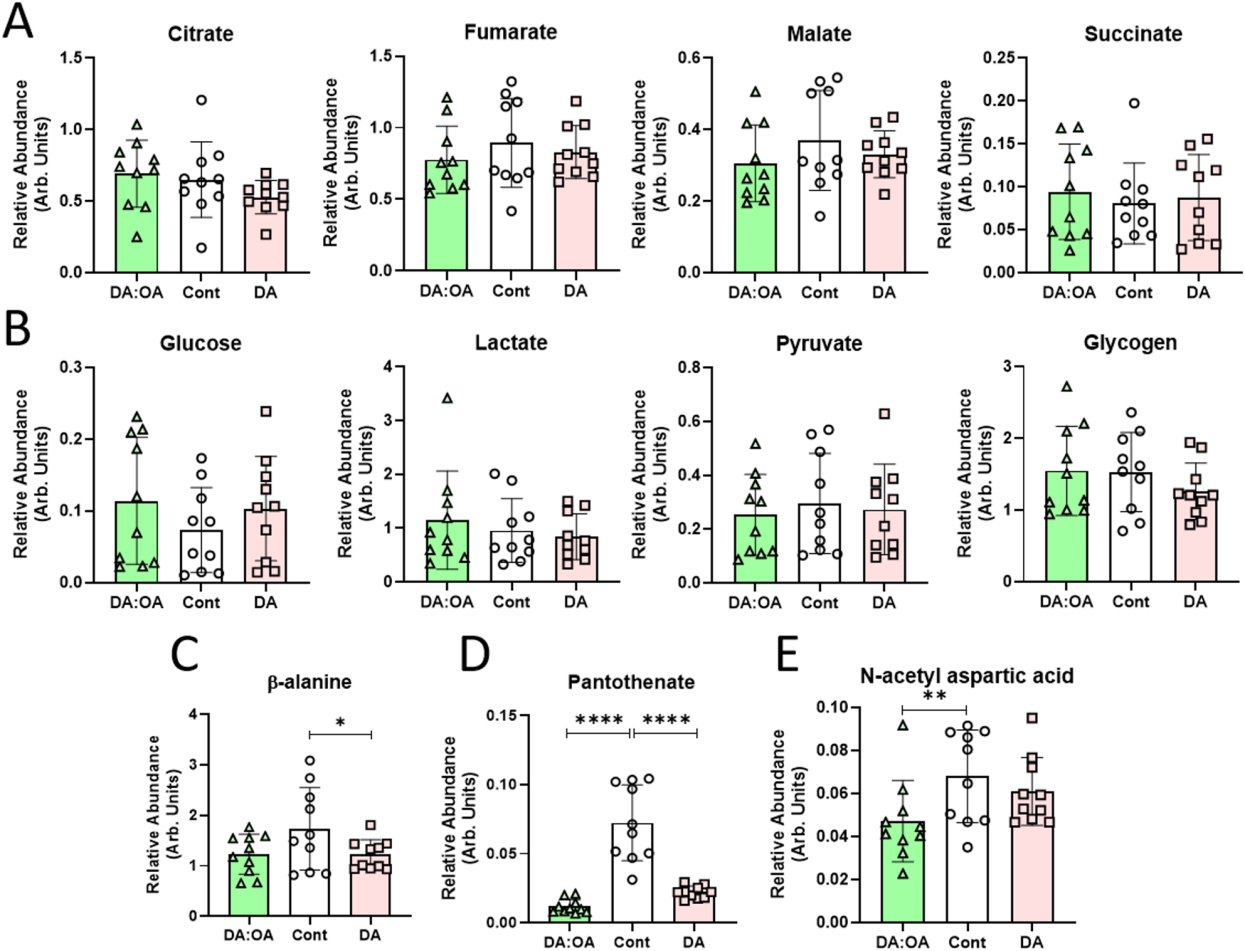
Metabolomic analysis of GBM cells following MCFA treatment. Cells were treated with decanoic acid (150 µM) or DA: OA (120:30 µM decanoic: octanoic acid) at a total concentration of 150 µM, for 4 days, using 10 independent samples (*n* = 10). Polar and protein/lipid extracts were analysed by GC-MS and quantified. **A **TCA cycle related metabolites, citrate, fumarate, malate and succinate. **B** Energy related metabolites, glucose, lactate, pyruvate and glycogen. **C **β-alanine, **D** Pantothenate, **E** N-acetyl aspartic acid. * *P* < 0.05, ** *P* < 0.01, ****P < 0.0001

Metabolic mechanisms for decanoic acid and DA: OA were investigated using the same treatment regimen, focusing on potential energy-related changes that may indicate a therapeutic approach in cancer cell treatment [47]. From this analysis, DA: OA and decanoic acid treatment did not alter levels of TCA components (Fig. 5A) (Supplementary Fig S9), nor did they alter key energy molecules (glucose, lactate, pyruvate and glycogen; Fig. 5B). However, surprisingly, decanoic acid reduced β-alanine levels (*P* < 0.05) (Fig. 5C), and both treatments reduced pantothenate levels (vitamin B5, *P* < 0.0001) (Fig. 5D), which is an essential cofactor for the synthesis of coenzyme A [48] required for the use of fatty acids in energy metabolism [49]. This is important as GBM cells are reliant on fatty acid metabolism as an energy source [27]. Furthermore, N-acetyl aspartic acid is synthesised from acetyl CoA and aspartate (Fig. 5E), and is also significantly reduced following DA: OA treatment, where it functions as a cancer marker [50] and is elevated in some cancers [51].

## Discussion

Ketogenic diets provide a potentially exciting new approach for the treatment of refractory cancers where dietary glucose reduction and resulting ketone generation may provide an adverse environment for cancer progression [1]. However, a recently introduced MCFA dietary supplement that has provided therapeutic benefit in the treatment of epilepsy [18] without requiring carbohydrate restrictions or resulting ketosis has not been investigated for its molecular mechanism in cancer cells. Here we investigate the constituents of this new diet, decanoic and octanoic acid, and the DA: OA blend used clinically [18], to regulate cellular energy-related pathways in human GBM-derived cells [23, 24]. These fatty acids are brain penetrant, with a brain to plasma ratio up to 0.79 ± 0.16 [52], and octanoic acid may function to reduce the metabolism of decanoic acid [53]. Molecular analysis identified distinct effects of the individual fatty acids and the DA: OA blend, with dominant effects of decanoic acid and DA: OA, indicating structural specificity of cellular effects that are unlikely to be caused by fatty acid metabolism alone, and that these components may trigger key molecular effects relating to cancer cell biology.

Under physiological conditions, glucose catabolism is preferentially used as an energy source via glycolysis in cancer cell proliferation [54] and is significantly upregulated in GBM tumours, providing a potential therapeutic target [26]. We show that GBM cells exposed to decanoic acid-containing treatments, even at high glucose levels [55], significantly downregulate expression of genes in multiple pathways including glycolysis, other carbohydrate metabolic pathways, and the Hypoxia Inducible Factor 1 (HIF-1) signalling pathway. The HIF-1 pathway triggers glycolysis and lactate metabolism, decreases mitochondrial respiration, promotes angiogenesis and increases cell proliferation in cancer [56], suggesting that a downregulation of the pathway is consistent with a potent anti-cancer mechanism [57]. KEGG pathway analysis of proteomic changes confirmed the regulation of similar pathways, including metabolic, glucose, cancer, and kinase pathways following DA: OA treatment.

β-oxidation provides an essential role in cell energy metabolism, where fats are activated by coenzyme A and used to supply ATP through oxidative phosphorylation. The canonical mechanism of ketogenic diets is through the use of ketones as energy source to feed oxidative phosphorylation [58], where these diets reduce growth of various cancers with deficiencies in this energy pathway [2]. Astrocytes are capable of metabolising MCFAs where neurons cannot [59], and GBM-derived cell lines show enhanced transcription of fatty acid metabolism [27], a finding that if further supported here. The increased expression of fatty acid metabolism enzymes such as carnitine palmitoyl transferase A1 (CPT1A) and acyl-CoA dehydrogenase very long chain (ACADVL) has also been shown in human-derived fibroblasts following 6 day decanoic acid treatment (250 µM) [60], and these changes are confirmed in our data. Thus, MCFA treatment of GBM cells triggers increased expression of fatty acid metabolism genes, likely to enhance energy provision.

Surprisingly, metabolomic analysis of GBM-derived human cells identified an unexpected mechanism of both decanoic acid and DA: OA to reduce pantothenate levels. Pantothenate is a vitamin and is an essential component of coenzyme A, necessary for fatty acid-derived energy provision, and it is rapidly absorbed into plasma following dietary supplementation [61]. It is transported via a sodium-dependent multivitamin transporter (SMVT) [62] that is also responsible for biotin and alpha-lipoic acid uptake. Since the transcriptional changes identified here may enhance the use of fatty acids as energy source, the reduction in pantothenate following MCFA treatment may limit fatty acid utilisation by constraining coenzyme A availability. This depletion may also be enhanced by elevated biotin or alpha-lipoic acid levels [61], or through potential SMVT inhibitors [63]. Reduced β-alanine levels caused by decanoic acid treatment may also reflect mechanisms that could inhibit tumour growth [64]. Hence, future clinical trials of MCFA in GBM therapy may consider avoiding pantothenate-containing supplements to maximise therapeutic effects or investigate approaches to reduce cellular pantothenate levels.

Insulin signalling has also been linked to both ketogenic diets and cancer treatments. These diets reduce insulin levels [65] or decrease insulin requirement [66], and the enhancement of insulin/IGF system and downstream PI3K/AKT/mTOR pathway are associated with cancer progression [67]. One effect of the ketogenic diet is attributed to a reduction in glucose and insulin signalling, leading to a decrease in mTORC1 activation [68]. Data presented here indicate that decanoic acid treatment may reduce insulin signalling [65]. In addition, these data indicate a reduction in the expression of the mTORC1 substrate 4EBP1, which may provide a mechanism of downregulating this pathway in support of recent studies [17]. These observations may provide evidence for the efficacy of decanoic acid in cancer treatment, but also in diabetes, in the absence of glucose restrictions [69].

Some of the mechanisms for MCFAs shown here are supported by related studies, although without analysis of the specific blend shown to be clinically effective in epilepsy treatment [18, 70], and following acute treatment time where metabolic changes have not taken effect [25]. Using hepatocellular carcinoma (HCC) cells [71], 24 h decanoic acid treatment provided an acute reduction in kinase-dependent phosphorylation of the Hepatocyte Growth Factor receptor, that regulates kinase various kinases including PI3K and MAPK [72], consistent with that shown by the clinically effective MCFA blend. Another study in a distinct GBM cell line (U87MG) [9], again using 24 h treatment at above physiological levels (300 µM), confirmed acute and distinct mechanisms for MCFAs with changes in TCA cycle and energy metabolites not seen in this current study (following chronic treatment), suggesting adaptive changes in cancer cell biology over time.

As the main purpose of this study was to investigate the potential therapeutic effect of the MCTKD on cancer, one limitation of the experimental design is the focus on the effects of decanoic and octanoic acid without including long chain fatty acids (LCFAs), present in the classic ketogenic diet. LCFAs provide fuel for β-oxidation, and palmitate has been shown to replenish the TCA cycle in endothelial cells [73]. Comparison between MCFAs and long chain fatty acids in this context therefore warrants further investigation. In addition, further studies could examine resulting physiological changes following treatment and monitor pantothenate levels in future clinical settings.

Thus, this study investigates the cell and molecular mechanisms of a new dietary approach, used in the treatment of patients with drug resistant epilepsy [18], in human GBM cells. By employing clinically relevant levels of the dietary constituents, and allowing time for metabolic changes [25], the study identifies a range of cellular changes with potential impact on cancer development. The study indicates metabolic reprogramming at a transcriptional level by decanoic acid containing treatments, leading to a reduction in glucose metabolism and an increase in fatty acid metabolism. The study also identifies protein level changes associated with enhanced fatty acid uptake, through elevated albumin [37], and a network of signalling pathways including kinases and RAS targeting. Crucially, the study suggests that metabolic reprogramming of GBM cells leads to a deficit in pantothenate levels, a key component of fatty acid metabolism, suggesting potential clinical benefit for avoiding pantothenate supplements during MCTKD and other ketogenic diets to limit energy supply to proliferating cancer cells [26, 27].

## Supplementary Information

Supplementary Material 1.

## Data Availability

The dataset supporting the conclusions of this article is included PRIDE repository (PXD068251) ([https://www.ebi.ac.uk/pride/] (https:/www.ebi.ac.uk/pride)) or as additional files. Reviewer access is via the PRIDE website, project access: PXD68251, TOKEN: XMpGhpsltl5Z.
