# Supplementary material for: Metabolic targeting of pantothenate by medium chain fats in glioblastoma cells
Source: BMC Cancer. 2026 Apr 7;26:621. doi: 10.1186/s12885-026-15909-3 (PMC13181877; doi:10.1186/s12885-026-15909-3)
Supplement: Supplementary file 1 — Supplementary Material 1. [file 12885_2026_15909_MOESM1_ESM.docx]

**Supplementary figures**

**Supplementary Fig S1:**


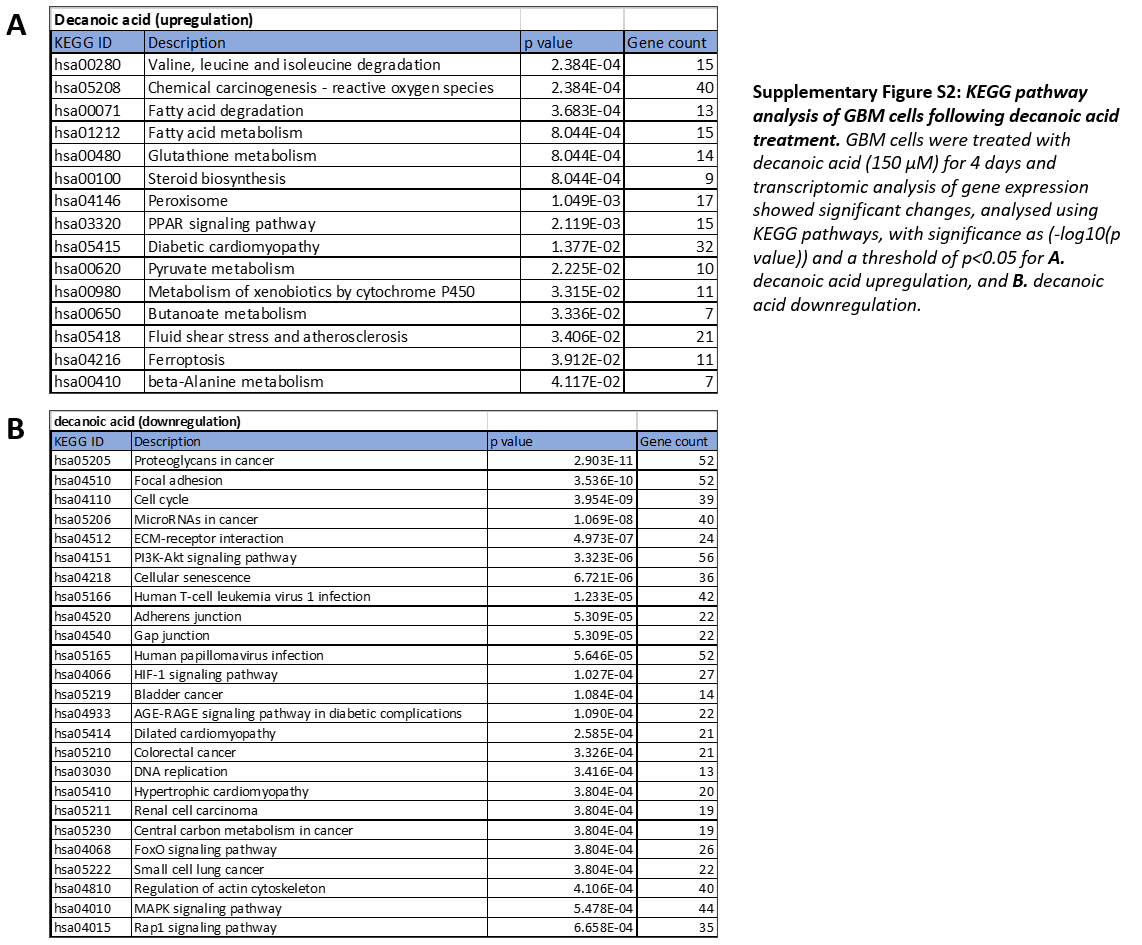


**Supplementary Fig S1:** KEGG pathway analysis of GBM cells following decanoic acid treatment. GBM cells were treated with decanoic acid (150 μM) for 4 days and trasnscriptomic analysis of gene expression showed significant changes, analysed using KEGG pathways, with significance as (-log 10(pvalue)) and threshold of p<0.05, for A. decanoic acid upregulation, and B. decanoic acid downregulation.

**Supplementary Fig S2:**


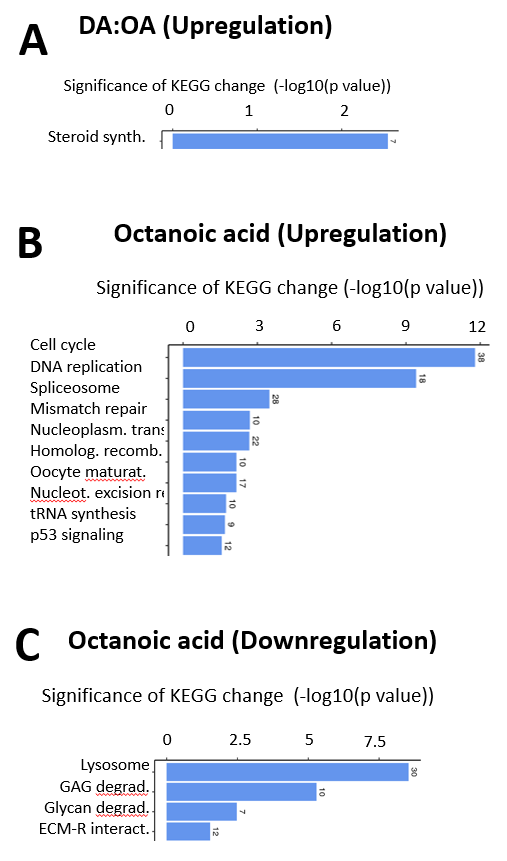


**Supplementary Figure S2: *Transcriptomic analysis of GBM cells following treatment with MCFAs.*** *GBM cells were treated with decanoic acid or octanoic acid (150 µM) or a Decanoic Acid Rich Supplement (DA:OA: 120 μM decanoic acid and 30 μM octanoic acid) for 4 days and transcriptomic analysis of gene expression showed significant changes, analysed using KEGG pathways, with significance as (-log10(p value)) with significance threshold of p<0.05.* ***A.*** *DA:OA treatment provide one KEGG pathway significantly upregulated.* ***B.*** *Octanoic acid treatment provided 10 KEGG pathways significantly upregulated, and* ***C.*** *four pathways significantly downregulated.*

**Supplementary Fig S3:**


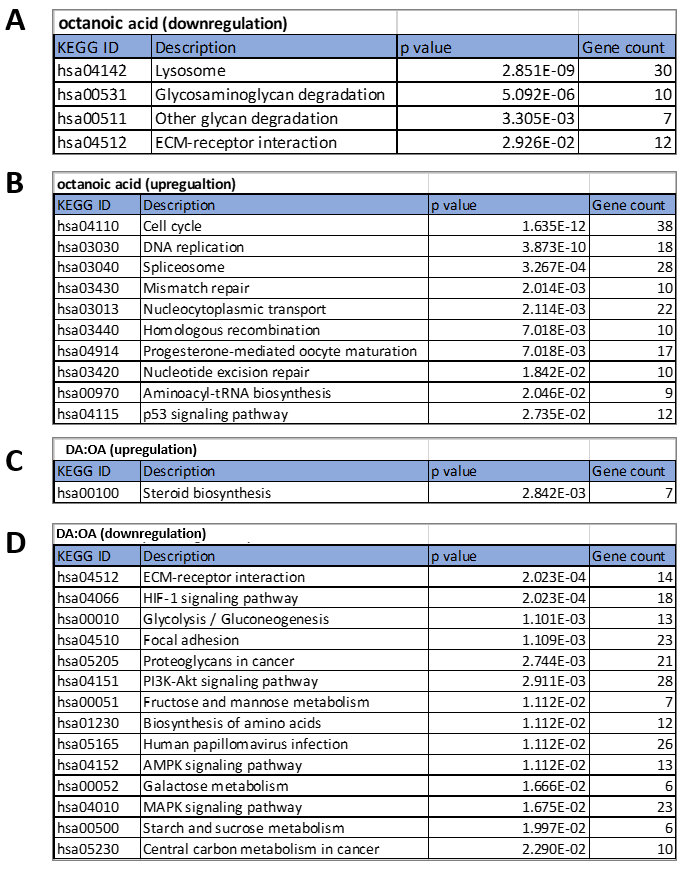


**Supplementary Figure S3: *KEGG pathway analysis of GBM cells following octanoic acid and DA:OA treatment.*** *GBM cells were treated with octanoic acid (150 µM) or a DA:OA (120 μM decanoic acid and 30 μM octanoic acid) for 4 days and transcriptomic analysis of gene expression showed significant changes, analysed using KEGG pathways, with significance as (-log10(p value)) with a threshold of p<0.05 for A. octanoic acid downregulation, and B. upregulation, and for DA:OA C. upregulation and D. downregulation.*

**Supplementary Fig S4:**


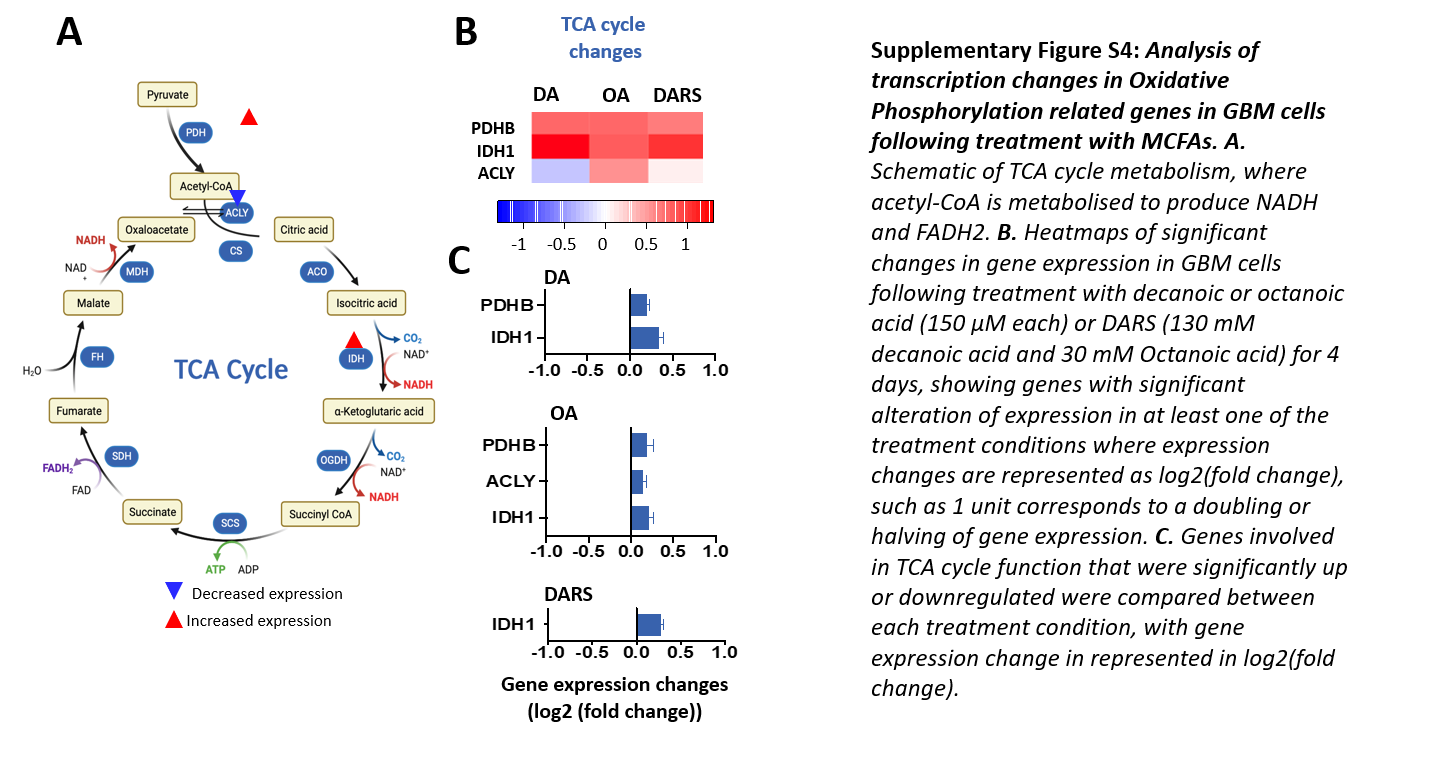

**Supplementary Figure S5:** Proteomic analysis (proteins named A-M) of cells following treatment with DA:OA or DA at a total concentration of 150 μM, for 4 days, and derived protein extracts analysed by LC-MS and quantified using PEAKS. Statistical analysis of the whole protein data set employ 2-way ANOVA with Dunnett's correction from n=4 independent samples, with each protein identified at least 3 times. * P<0.05, ** P<0.01, ***P<0.001, ****P<0.0001.

**Supplementary Figure S6:** Proteomic analysis (proteins named N-Z) of cells following treatment with DA:OA or DA at a total concentration of 150 μM, for 4 days, and derived protein extracts analysed by LC-MS and quantified using PEAKS. Statistical analysis of the whole protein data set employ 2-way ANOVA with Dunnett's correction from n=4 independent samples, with each protein identified at least 3 times. * P<0.05, ** P<0.01, ***P<0.001, ****P<0.0001.

**
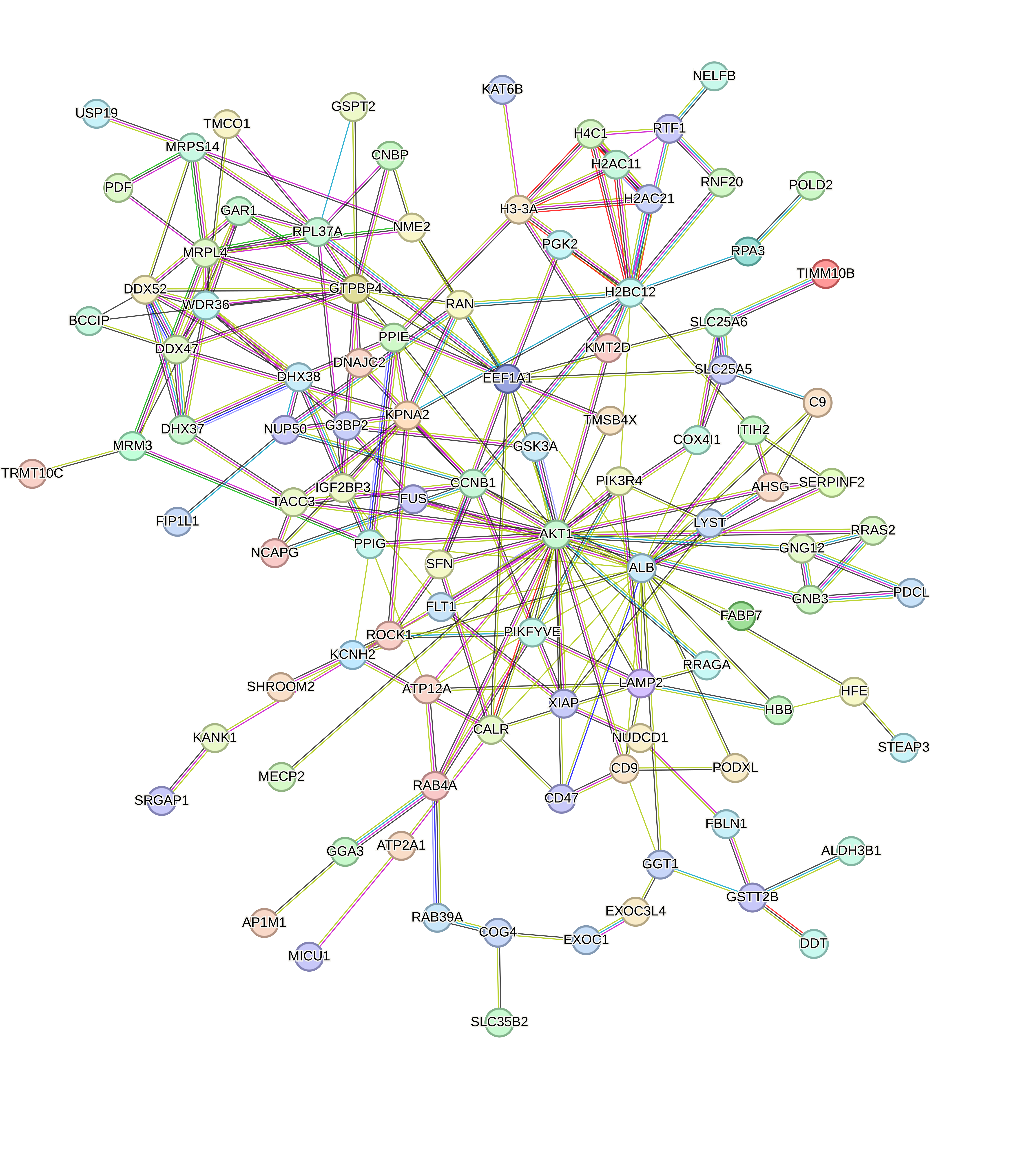
**

**Supplementary Figure S7:** String analysis proteins shown to significantly change following treatment with MCFAs. Cells were treated with DA:OA (decanoic and octanoic acid at an 80:20 ratio) or DA (decanoic acid only) at a total concentration of 150 μM, for 4 days, and derived protein extracts analysed by LC-MS and quantified using PEAKS. String analysis with orphan proteins and 2 degree links longer than two proteins were removed for clarity.

**
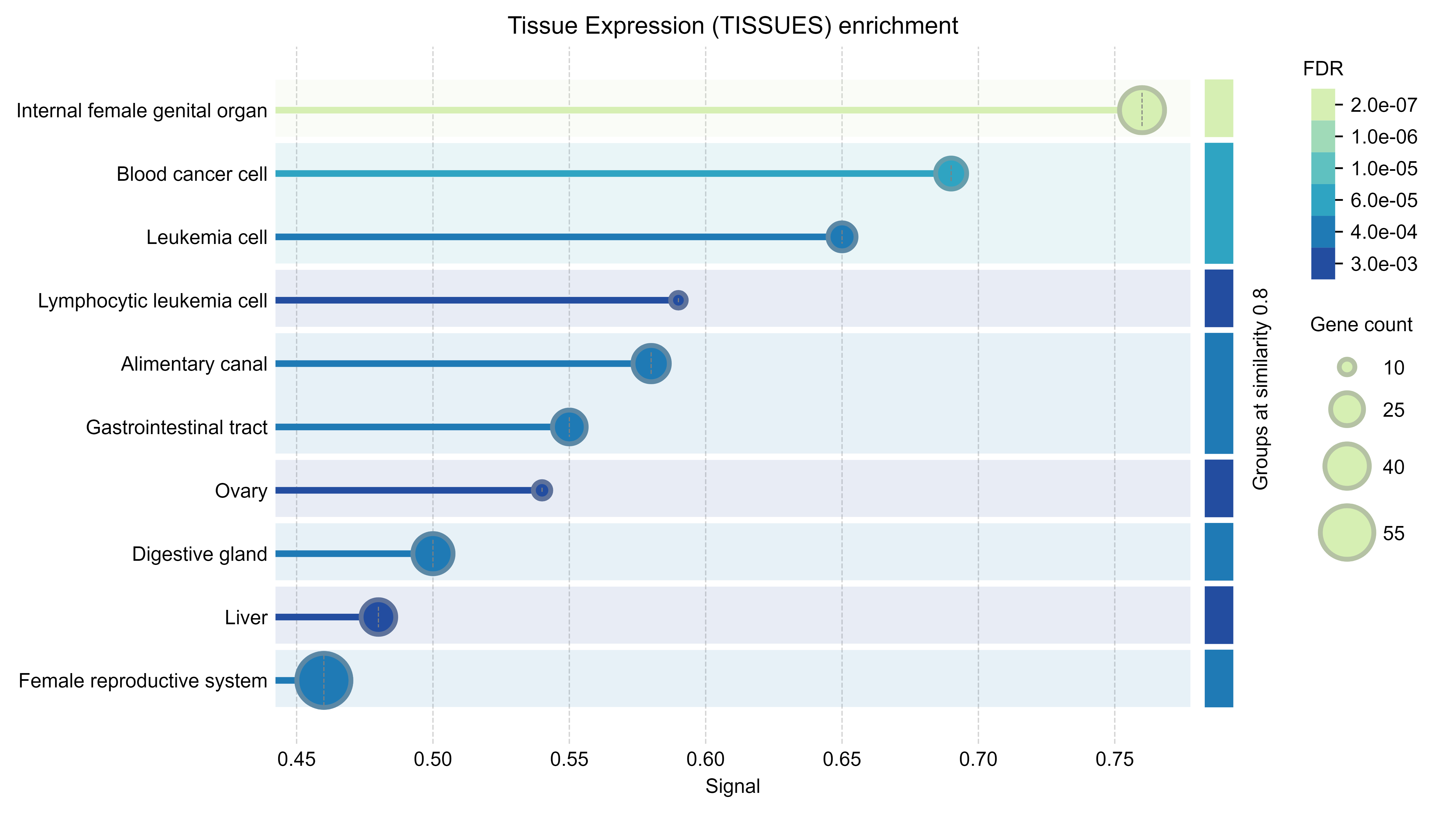
**

**Supplementary Figure S8:** GO analysis of tissue protein expression enrichment. Tissue specific changes in protein levels identified a range of cell types likely to be regulated with DA and DO:OA treatment.

**Supplementary Figure S9:** Metabolomic analysis following. Cells were treated with DA:OA (decanoic and octanoic acid at an 80:20 ratio) or DA (decanoic acid only) at a total concentration of 150 μM, for 4 days, and derived metabolic extracts were analysed. N=10.
